# Direct Effective Dose Calculations in Pediatric Fluoroscopy-Guided Abdominal Interventions with Rando-Alderson Phantoms – Optimization of Preset Parameter Settings

**DOI:** 10.1371/journal.pone.0161806

**Published:** 2016-08-24

**Authors:** Moritz Wildgruber, René Müller-Wille, Holger Goessmann, Wibke Uller, Walter A. Wohlgemuth

**Affiliations:** 1 Institut für Röntgendiagnostik, Universitätsklinikum Regensburg, Regensburg, Germany; 2 Institut für klinische Radiologie, Westfälische Wilhelms-Universität Münster, Universitätsklinikum Münster, Münster, Germany; North Shore Long Island Jewish Health System, UNITED STATES

## Abstract

**Objective:**

The aim of the study was to calculate the effective dose during fluoroscopy-guided pediatric interventional procedures of the liver in a phantom model before and after adjustment of preset parameters.

**Methods:**

Organ doses were measured in three anthropomorphic Rando-Alderson phantoms representing children at various age and body weight (newborn 3.5kg, toddler 10kg, child 19kg). Collimation was performed focusing on the upper abdomen representing mock interventional radiology procedures such as percutaneous transhepatic cholangiography and drainage placement (PTCD). Fluoroscopy and digital subtraction angiography (DSA) acquisitions were performed in a posterior-anterior geometry using a state of the art flat-panel detector. Effective dose was directly measured from multiple incorporated thermoluminescent dosimeters (TLDs) using two different parameter settings.

**Results:**

Effective dose values for each pediatric phantom were below 0.1mSv per minute fluoroscopy, and below 1mSv for a 1 minute DSA acquisition with a frame rate of 2 f/s. Lowering the values for the detector entrance dose enabled a reduction of the applied effective dose from 12 to 27% for fluoroscopy and 22 to 63% for DSA acquisitions. Similarly, organ doses of radiosensitive organs could be reduced by over 50%, especially when close to the primary x-ray beam.

**Conclusion:**

Modification of preset parameter settings enabled to decrease the effective dose for pediatric interventional procedures, as determined by effective dose calculations using dedicated pediatric Rando-Alderson phantoms.

## Introduction

The use of medical imaging and intervention in children has steadily increased over the last decades. Most data regarding radiation dose in children is derived from Computed Tomography (CT) examinations. Although CT is considered the greatest source of medical radiation exposure, a 2009 report on radiation dose emphasized the increasing pediatric dose from fluoroscopy-guided interventional procedures together with the need to promote radiation safety precautions[1]. Ultrasound is the preferred modality for pediatric image-guided therapies as it avoids radiation exposure. However, for many pediatric interventions fluoroscopy and digital subtraction angiography (DSA) are still required[2]. Although technical advances and increased knowledge on radiation exposure have led to a substantial decrease of radiation dose, especially long and complex procedures still carry the likelihood of higher and potentially harmful radiation doses. For neurovascular interventions the radiation dose associated with fluoroscopic and angiographic imaging carries an inherent risk to pediatric patients[3]. Children are even more susceptible to the adverse effects of ionizing radiation compared to adults.

Patient dose is best reported in form of the effective dose as this parameter allows comparison of radiation burden between various imaging techniques and similarly between different procedures. Effective dose calculations for x-ray-guided interventional procedures are difficult due to different acquisition modes (fluoroscopy versus DSA), various acquisition angles, collimation, table positions and magnifications. There are various ways to estimate the effective dose for interventional procedures. Monte-Carlo simulations can help to calculate a theoretic effective dose by following the statistically probable path of the x-ray beam as it passes through and deposits energy in a standard set of electronically generated phantoms[4]. A much more precise, direct method to determine the effective dose in interventional radiology procedures is the use of multiple thermoluminescent dosimeters (TLDs) placed at various locations representative for different organs in an anthropomorphic phantom[5]. The organ doses from multiple locations within the phantom are subsequently directly measured to calculate the effective dose for the entire procedure. Preset parameters for dedicated pediatric fluoroscopy and DSA programs provided by the manufacturer may be empirically determined and not well scientifically validated and may still carry the potential for further dose reduction.

Specific aim of the current study was to 1) use pediatric phantoms to directly measure the effective dose for abdominal interventions using standard acquisition parameters as provided by the manufacturer and 2) compare the calculated effective doses after adjustment of the acquisition parameters to evaluate the potential for further dose reduction in pediatric interventional procedures. Our hypothesis was that the default acquisition parameters for pediatric interventions can be substantially modified resulting in a decreased effective dose for fluoroscopy and DSA.

## Materials and Methods

### Pediatric Phantoms

Direct effective dose measurements were performed using three anthropomorphic Rando Alderson phantoms (Radiology Support Devices Inc., CA, USA)[5]:

- Newborn (‘Stanley’): a 3.5kg phantom representing a mature newborn.
- Toddler (‘Clifford’): a 10kg phantom representing a small kid at 1–3 years.
- Child (‘Braden’): a 19kg phantom representing a child of 3–7 years.

Phantoms consist of natural human bones embedded in soft tissue equivalent material. Absorption and scattering of the phantom are equivalent to those in humans. Each phantom is divided in transverse sections of 2.5cm slice thickness, each incorporating a grid of holes in z-direction, which incorporates the TLDs perpendicular to the radiation beam. TLDs are embedded in acrylic glass holder tubes as provided by the manufacturer. TLDs are placed within each slice according to the specific anatomical location of each organ/tissue for calculation of the effective dose. In total, 96 TLDs are placed in the newborn phantom, 140 TLDs in the toddler, and 146 TLDs in the child phantom. All organs important for effective dose calculations were equipped with at least three TLDs. Standard lithium-fluoride TLDs (TLD 100 rods, 1x1x6mm, Harshaw, Cleveland, OH) were used as previously described[6]. The organ dose has been defined as the mean of the TLDs at each organ location. TLDs used in this study are accurate within a range of 10^-12^—10Gy, according to the manufacturer. To ensure that results remain stable and to estimate the variation of the TLDs, reference measurements are performed twice a year at our institution. For calibration of the TLDs according to different beam energies (70-150kV), an ionization chamber was applied (PTW, Freiburg, Germany). Calibration of the ionization chamber, which was performed before each new measurement, (according to the guidelines of the German calibration service DKD) avoids energy and beam quality dependency of the TLDs. After reading of the TLDs, the complete information from the previous measurement was cleared by heating the TLDs within an annealing oven (PTW, Freiburg, Germany). Immediately after clearance TLDs were placed within the phantoms for subsequent measurements. After radiation exposure TLDs were read out in a Harshaw reader (Harshaw, Cleveland, OH, USA) within 6h to avoid fading. Considering all potential causes of error, accuracy of the measurements is ± 10%[7–9]. For calculation of the effective patient dose, tissue-weighting factors according to the ICRP Publication 103 were applied, assuming a radiation-weighting factor of 1.0 for x-ray irradiation[10]. Effective dose (in mSv) was calculated as E = ∑W_Ti_*H_i_ where W_Ti_ is the tissue-weighting factor of individual organs, H_i_ = W_Ri_ * D_i_ represents the equivalent dose of individual organs, W_Ri_ is the radiation weighing factor (W_Ri_ = 1 for x-ray), and D_i_ is each individual organ dose.

### Angiography equipment

Both fluoroscopic and angiographic acquisitions of the three pediatric phantoms were performed on an Artis Zee biplane (Siemens Healthcare GmbH, Forchheim, Germany) angiography unit, equipped with a state of-the-art flat panel detector (30x40cm).

### Acquisition protocols

**Fig 1:**
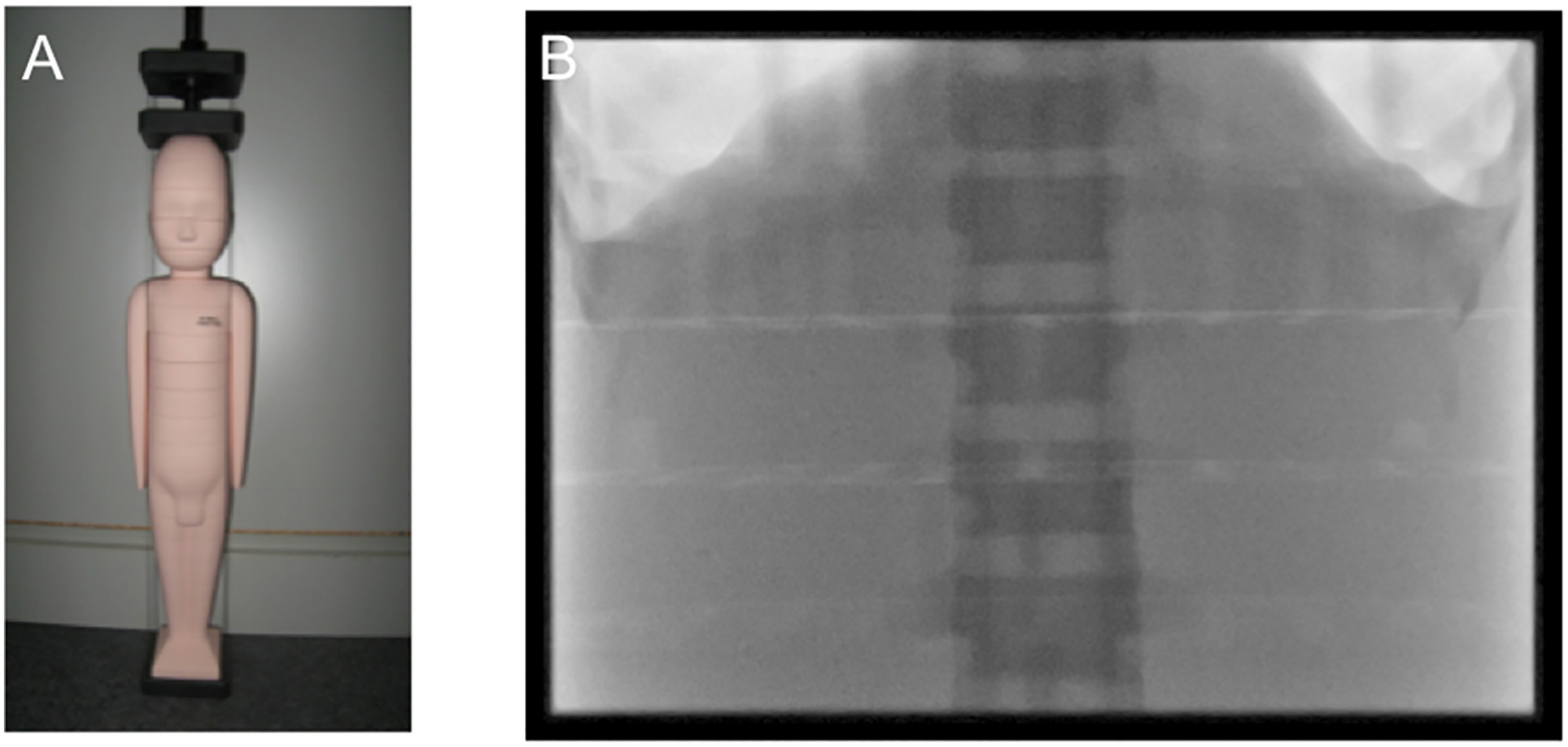
Anthropomorphic phantom and collimation. Panel (A) shows example of the smallest anthropomorphic phantom, representing a newborn with 3.5 Kg body weight. Panel (B) shows collimated field of irradiation exposure covering the upper abdomen and adjacent structures.

Collimation on the pediatric phantoms was set to cover the area of the upper abdomen (Fig 1), to mimic biliary or other pediatric abdominal interventions such as portal vein angioplasty. Organs in the primary field of view included liver, spleen, stomach, small bowel, colon, lower lung and bone marrow. Collimation was kept constant during all measurements for each phantom, however the collimation was adjusted between the different phantoms to cover the same area and include the same organs in the primary beam between all three phantoms. Acquisitions were performed monoplane in a strict poster-anterior geometry, scatter grid was applied only for the 19kg pediatric phantom. A copper (Cu) filtration of 0.9mm was applied for each phantom. The source image distance was set to 100cm for the child and 120cm for the toddler and newborn phantom.

To match typical clinical applications, fluoroscopy was performed for a continuous time period of 15 minutes on each phantom with a frame rate of 7.5 f/s to acquire enough dose for subsequent comparisons. Each pediatric phantom was examined with fluoroscopy before and after modification of the preset parameters. DSA series were acquired for continuous 5 minutes with a frame rate of 2 f/s for each phantom. Again, each pediatric phantom was examined before and after modification of the preset parameters. Modification of the default parameters was performed by lowering the entrance dose at the detector side, and was carried out under close agreement between the manufacturer and the investigators, respectively the interventional radiologist. Standard as well as modified acquisition parameters for fluoroscopy and DSA acquisitions are presented in Table 1.

**Table 1:** Standard and modified pediatric acquisition parameters for both fluoroscopy and DSA. DAP = Dose Area Product, Entr. Dose = Entrance Dose (of the detector).

	Fluoroscopy (15 Min)		DSA acquisitions (5 Min)	
Phantom	Preset parameters	Modified parameters	Preset parameters	Modified parameters
**Newborn 3.5k**g				
Tube Voltage (kV)	70	70	66	66
Entr. Dose (nGy/pulse)	29	18	1200	540
Air Kerma Dose (mGy)	4.2	2.6	2.9	1.3
DAP (μGy*m^2^)	21.98	18.21	22.38	9.5
Scatter Grid	No	No	No	No
Frame/sec	7.5	7.5	2	2
**Toddler 10kg**				
Tube Voltage (kV)	70	70	66	66
Entr. Dose (nGy/pulse)	29	18	1200	540
Air Kerma Dose (mGy)	8.1	5.4	5.8	3.0
DAP (μGy*m^2^)	73.17	50.4	68.13	34.86
Scatter Grid	No	No	No	No
Frame/sec	7.5	7.5	2	2
**Child 19kg**				
Tube Voltage (kV)	70	70	66	66
Entr. Dose (nGy/pulse)	29	18	1200	540
Air Kerma dose (mGy)	7.8	5.3	6.0	2.9
DAP (μGy*m^2^)	114.23	86.24	112.14	51.7
Scatter Grid	Yes	Yes	Yes	Yes
Frame/sec	7.5	7.5	2	2

To evaluate the resulting image quality in the phantoms investigated, three independent readers independently assessed the subject image quality with respect to signal-ti-noise ratio following a four-point Likert scale: 4 = excellent image quality/high signal-to-noise, 3 = good image quality/acceptable signal-to-noise, 2 = impaired image quality/low signal-to-noise, 1 = poor image quality/non-diagnostic signal-to-noise. Phantom measurements were performed in singlet only, so no statistical analysis was conducted.

## Results

Values for the effective dose were calculated from the measurements for 15 minutes fluoroscopy as well for 5 minutes DSA acquisition before as well as after modification of the preset parameters. Results were calculated to a standardized irradiation time of one minute and as such presented in Table 2. Modification of preset parameters enabled an effective dose reduction of 12 to 27% for fluoroscopy and 22 to 63% for DSA acquisitions according to the different size of the pediatric phantoms. Similarly Air Kerma Dose was substantially decreased between 32 to 38% for fluoroscopy and 48 to 55% for DSA acquisitions.

**Table 2:** Measured effective dose for standard and modified acquisition parameters.

	Fluoroscopy			DSA acquisitions		
Phantom	Preset parameters	Modified parameters	% change	Preset parameters	Modified parameters	% change
Newborn (3.5kg)	0.011	0.009	18	0.14	0.11	22
Toddler (10kg)	0.025	0.022	12	0.60	0.22	63
Child (19kg)	0.049	0.036	27	0.65	0.31	52

Effective dose values are given in mSv, calculated for a fluoroscopy or DSA duration of 1 minute.

Organ doses for both fluoroscopy as well as DSA acquisitions before and after modification of preset parameters are presented in Table 3. Adjustment of preset parameters allowed for organ dose reductions of 0 to 63% for fluoroscopy and 0 to 76% for DSA acquisitions for the various organs. As expected, the highest potential of dose reduction was achieved in organs directly located within the irradiation field, respectively the liver.

**Table 3:** Organ dose values for standard and modified acquisition parameters.

	Fluoroscopy			DSA acquisitions		
Phantom	Preset parameters	Modified parameters	% change	Preset parameters	Modified parameters	% change
Newborn (3.5kg)						
Lung	0.004	0.002	50	0.049	0.040	18
Esophagus	0.000	0.000	-	0.002	0.002	-
Liver	0.001	0.001	0	0.010	0.010	0
Stomach	0.001	0.001	0	0.018	0.014	22
SB	0.000	0.000	-	0.001	0.001	-
Colon	0.000	0.000	-	0.010	0.005	50
Testes	0.001	0.000	-	0.002	0.001	50
Uterus	0.000	0.000	-	0.000	0.000	-
BM	0.001	0.000	-	0.003	0.002	33
Toddler (10kg)						
Lung	0.005	0.004	20	0.105	0.042	60
Esophagus	0.000	0.000	-	0.005	0.003	40
Liver	0.001	0.001	0	0.060	0.014	76
Stomach	0.004	0.004	0	0.124	0.046	63
SB	0.000	0.000	-	0.012	0.004	66
Colon	0.003	0.002	33	0.065	0.022	66
Testes	0.000	0.000	-	0.002	0.002	0
Uterus	0.000	0.000	-	0.000	0.000	-
BM	0.000	0.000	-	0.014	0.006	57
Child (19kg)						
Lung	0.008	0.005	37	0.118	0.042	64
Esophagus	0.001	0.000	-	0.021	0.005	76
Liver	0.011	0.003	63	0.047	0.025	47
Stomach	0.012	0.009	25	0.139	0.064	54
SB	0.000	0.000	-	0.004	0.003	25
Colon	0.004	0.003	25	0.066	0.033	50
Testes	0.000	0.000	-	0.001	0.001	0
Uterus	0.000	0.000	-	0.000	0.000	-
BM	0.011	0.008	27	0.126	0.072	43

Organ dose values are provided in mSv, presented for a fluoroscopy or DSA duration of 1 minute. SB = small bowel, BM = red bone marrow. % change of organ dose is provided wherever reasonable, otherwise no value is presented (-).

Image quality of the phantom scans, as judged by three independent readers is presented in Table 4.

**Table 4:** Subjective imaged quality, evaluated by three independent readers following a four-point-Likert scale: 4 = excellent image quality/high signal-to-noise, 3 = good image quality/acceptable signal-to-noise, 2 = impaired image quality/low signal-to-noise, 1 = poor image quality/non-diagnostic signal-to-noise.

**Fluoroscopy**			
Reader	Newborn	Toddler	Child
1			
Before adjustm.	4	3	3
After adjustm.	3	3	3
2			
Before adjustm.	4	4	3
After adjustm.	3	3	2
3			
Before adjustm.	4	4	3
After adjustm.	4	2	2
**DSA**			
Reader	Newborn	Toddler	Child
1			
Before adjustm.	4	4	3
After adjustm.	3	3	3
2			
Before adjustm.	3	3	3
After adjustm.	3	3	2
3			
Before adjustm.	3	4	3
After adjustm.	3	3	3

## Discussion

Radiation exposure should be as low as reasonable achievable (ALARA concept) in any procedure applying x-rays[11]. Calculation of the effective dose is a precise parameter for the evaluation of diagnostic and interventional imaging procedures. The relation between CT scanning and the increased risk of cancer has been well established for the pediatric population[12]. For interventional fluoroscopic procedures the risk association for repeated radiation exposure is less clear. Highest effective dose levels are reached during pediatric interventions for congenital heart disease, as these procedures require an exceptionally high frame rate and long fluoroscopy and DSA acquisition times[13, 14]. Apart from data dealing with pediatric cardiac intervention patients only very few studies are available investigating radiation exposure during other pediatric interventional radiology procedures. Pediatric patients requiring abdominal interventions, e.g. pediatric liver transplant recipients presenting with portal vein or biliary disease usually require repeated interventions leading to an accumulated risk[15, 16]. The life expectancy of patients with pediatric liver transplant is reduced compared to the normal population[17], however other pediatric interventional procedures such as embolization of extracranial vascular malformations involve children with a normal life expectancy. Especially in those, the reduction of stochastic radiation risk may be relevant.

We performed a thorough investigation of the entrance dose during abdominal interventional procedures using dedicated pediatric anthropomorphic phantoms, representing children at various age[5]. Each phantom was equipped with multiple TLDs that enabled reliable measurements of organ dose and subsequent calculation of the effective dose applying the IRCP 103 weighting factors[10]. TLDs were calibrated before each measurement. For fluoroscopy, effective dose values remained constantly below 0.1mSv per minute fluoroscopy time. For DSA acquisitions entrance dose values remained below 1mSv per minute DSA acquisition applying a frame rate of 2 f/s (respectively 120 frames per minute). Those levels are up to two orders of magnitude lower compared to pediatric cardiology interventions[14, 18–20]. Although already in a low range, we sought to further reduce radiation exposure by modifying preset parameters of standardized acquisition programs as provided by the manufacturer. Modification of preset parameters, performed in agreement between the manufacturer and the investigators, enabled a reduction of the effective dose from 12 to 27% for fluoroscopy and 22 to 63% for DSA acquisitions. Similarly single organ doses were substantially reduced by modification of the preset parameters, which was most evident for organs included in the primary x-ray beam. As the precise effective dose can only be calculated using anthropomorphic phantoms, many institutions use Air Kerma Dose to determine the amount of irradiation applied to a patient. Air Kerma Dose in our study similarly decreased substantially upon modification of the detector entrance dose, more prominent for DSA acquisitions, as expected. As we adapted the field of view for each phantom size to cover the same anatomical area of the upper abdomen, the change in dose area product was not proportional to the air kerma change.

In a study using pediatric anthropomorphic phantoms Miksys et al. showed that abdominal interventions carry the highest effective dose burden compared with chest and head/neck mock interventional procedures[21]. Effective dose results were in a range of 0.14 to 0.31mSv per minute of fluoroscopy in posterior-anterior beam direction, which is about one order of magnitude higher compared to our results. Yet, those acquisitions were performed using an image-intensifier system, which has been shown to produce higher radiation dose compared to flat-panel detector systems[16]. Unfortunately it was not mentioned if a scatter grid has been used. We applied a scatter grid, comparable to our clinical practice, for irradiation exposure of the 19kg pediatric phantom, while for the 3.5 and 10kg phantom the grid was removed. The grid is usually not necessary for children<15kg, as the amount of scatter radiation is limited. Cortis et al. have demonstrated that removal of the antiscatter grid during routine biliary interventions in a pediatric patient population significantly reduced patient radiation exposure with acceptable image quality[15]. Image quality of the acquired phantom scans did not reveal a substantial loss in image quality, as subjectively assessed b three independent readers. Of course, image quality assessment of the phantoms scans has only limited value, and image quality before and after parameter adjustment needs to be evaluated subsequently in vivo. We will assess image quality subsequently in real pediatric patients investigated with the modified parameter settings and compare the image quality with that of patients investigated with the default parameter setting before the adjustments have been performed.

Image quality, besides subjective impression, is generally judged using some kind of signal-to-noise (SNR) ratio. The higher the x-ray dose, the better the soft tissue resolution becomes as a result of improved SNR. For interventional radiology procedures a lower SNR can be frequently accepted, as a high soft tissue contrast is primarily needed for diagnostic procedures. For interventional procedures instead, a higher noise level can be frequently accepted without impairment of the trackability of guidewires, catheters and other interventional material.

Assessment of the effective dose using real measurements is superior over theoretical models such as Monte-Carlo simulations, although the difference between those model calculations and measured effective dose levels has been reported to be less than 10%[22]. However, the use of TLDs also carries some shortcomings. The variation of the sensitivity of the TLDs requires a thorough calibration process. Although TLDs don’t show an aging process, reference measurements are carried out twice a year at our institution to remove TLDs that do not longer maintain the necessary quality. Besides ruling out measurement errors due to incomplete annealing of the TLDs, each organ was assessed by multiple TLDs (at least three). Additionally the time between annealing, measurement and read-out was minimized to rule out a potential exposure to heat that may alter the measurements results. Impurities on the surface of the TLDs may lead to tribo- and chemiluminescence effects. Rinsing the space between TLD and photomultiplier by inert, oxygen-free gas is used to minimize those effects. According to these shortcomings, the accuracy of the measurements is ± 10%, which is well accepted.

Modification of the detector entrance dose is only one approach to reduce effective dose applied to the patient. Proper coning, table height adjustments, using fluoroscopy zooming instead of regular magnification, restriction of angled tube positions further enable relevant dose reduction. Those were not included in order not to increase the complexity of the data to a point where interpretation of the effect of each of those possible modifications cannot be judged separately from each other. Additionally, replacement of fluoroscopic guidance for certain steps of a particular intervention by ultrasound guidance can additionally reduce the amount of the effective dose and should be considered wherever possible.

Limitations of the work include that fact that phantom measurements have been performed as singlets due to the laborious and expensive procedure of subsequent TLD analysis. This limits the analysis of the data, not allowing adequate statistics. Although the data were in the range we expected from similar pediatric phantom dose measurements, a measurement error of the singlet assessments cannot be ruled out. Additionally, modification of dose parameters has to be evaluated with respect to the resulting image quality in vivo. Therefore image quality before and after the described adjustment of dose parameters will be assessed in a pediatric patient cohort undergoing hepato-biliary interventions and the resulting image quality will be compared to the dose reductions that will be achieved.

## Conclusions

In summary we could show that applying a state of the art flat-panel detector system the effective dose for abdominal interventions in a pediatric phantom model is lower as previously reported. Additional modifications of preset parameters enable further dose reduction, which is beneficial both for children requiring multiple interventions and the interventional radiology personnel themselves. Clinical application of the modified parameters settings, which we have implemented after the successful phantom experiments will demonstrate if the image quality achieved with those low-dose programs is sufficient for pediatric interventional procedures.
